# Effect of Obesity on Left Ventricular Systolic and Diastolic Functions Based on Echocardiographic Indices

**DOI:** 10.7759/cureus.37232

**Published:** 2023-04-06

**Authors:** Shubhda Gade, Anagha V Sahasrabuddhe, Kajal A Mohite, Nandkishor J Bankar, Shilpa S Chaudhary, Parikshit A Muley, Pranjali P Muley

**Affiliations:** 1 Physiology, Datta Meghe Medical College, Datta Meghe Institute of Medical Sciences, Wardha, IND; 2 Microbiology, Jawaharlal Nehru Medical College, Datta Meghe Institute of Medical Sciences, Wardha, IND; 3 Radiology, Datta Meghe Medical College, Datta Meghe Institute of Medical Sciences, Wardha, IND; 4 Physiology, Jawaharlal Nehru Medical College, Datta Meghe Institute of Medical Sciences, Wardha, IND

**Keywords:** diastolic function, cardiovascular imaging, left ventricular dysfunction, cardiovascular disease, obesity

## Abstract

Background: Left ventricular systolic and diastolic functions are known prognosticators for cardiovascular morbidity. One of the significant risk factors for cardiovascular diseases is obesity. The objective of this study is to determine the effect of obesity on the systolic and diastolic functions of the left ventricle on the basis of echocardiographic indices.

Methods: 75 obese and 75 averagely built subjects were studied. They had no other comorbidities. The indices of echocardiography of systolic and diastolic function were taken and assessed using recent recommendations from the European Association of Cardiovascular Imaging and the American Society of Echocardiography.

Results: The volume indices of systolic and diastolic function (ejection diastolic volume (EDV) and ejection systolic volume (ESV)) and iso-volumetric relaxation time (IVRT) showed a significant increase in obese subjects (p<0.05); however, the relative thickness of the wall and internal diameter were comparable to non-obese subjects. The indices of contractility like ejection fraction, early diastolic filling velocity and late diastolic filling velocity (E/A) ratio, and mitral annular velocity were significantly lower in the obese subjects as compared to non-obese subjects. It was also found that left atrial diameter in systole and diastole had a moderate association (r=0.48, P<0.0001; r=0.35, P<0.0005) while mitral inflow E/A ratio had a negative association with body mass index (BMI) (r=−0.26, P=0.0166).

Conclusions: Volumetric changes and ejection are significantly altered by increased BMI. More comprehensive studies in the future are recommended to assess the same.

## Introduction

Obesity is a global health issue as it is constantly increasing in worldwide prevalence, from 4.6% in 1980 to 14.0% in 2019; while in India, its prevalence was reported to vary from 11.8% to 31.3% [1,2]. It is a complex condition and a major public health concern at present time because of its relationship with mortality; comorbidities such as cardiovascular diseases; and risk factors like hypertension, diabetes mellitus, and hyperlipidemia [1-3].

According to research, it is a lone predictor of eventual heart failure estimating up to 11% of all cases in men and up to 14% in women and accounts for about 45% of the increased risk of coronary heart disease [4,5]. Increased body mass index (BMI) has an irreversible effect not only on the structure but also on the function of the heart. The preload and afterload of the vascular structure are controlled by excessive body fat deposition because of peripheral resistance, hyperdynamic circulation, and chronic volume overload [6,7]. Additionally, it has been demonstrated that changes in adipose tissue composition might enhance leptin-related effects on blood pressure, which in turn causes left ventricular hypertrophy (LVH) [8]. As a consequence, both eccentric and concentric left ventricle (LV) geometric patterns are reported. LV dilatation and increased LV mass are frequent findings in obese patients [9-11].

As it impairs the LV's capacity to pump blood and fill with blood, left ventricular diastolic dysfunction (LVDD) has been linked in cohort studies to an increased risk of developing heart failure with a median prevalence rate of 11.8% (range of 4.7%-13.3%) in older adults [12-15]. Hence, attention is drawn to the pathophysiological relationships between fat and the potential development of heart failure. According to studies, LVDD in obese people is linked with structural abnormalities of the heart associated with cardiovascular disease [16,17]. However, the relationship between obesity and LVDD in absence of other systemic variables is rarely studied in the Indian population. Therefore, the current study’s objective was to compare the LV diastolic function of obese and non-obese persons on the basis of echocardiography findings.

## Materials and methods

A total of 150 apparently healthy subjects were selected for this study following approval from Institutional Ethical and Research Board, and informed written consent was obtained from all patients participating in the study.

The patients were categorized using the convenient stratified sampling method into two groups namely, Group A - obese (case) group and Group B - non-obese (control) group. The sample size was estimated based on the previous study by Chadha DS et al. in 2010 and considering the standard mean difference of ejection fraction (EF) (0.4%), power (80%), alpha error (5%), and a dropout rate of 10% [18].

Study population

Patients reporting for medical examination to the Datta Meghe Medical College, Nagpur, between August 2021 and May 2022 were screened.

Inclusion criteria

Patients above the age of 18 years were included.

Exclusion criteria

Pregnant females; patients with medical conditions like hypertension, diabetes mellitus, or dyslipidemia; patients with previous history or clinical evidence of coronary artery disease, heart failure, or cardiac valve disease; patients with respiratory disease; patients with the presence of chronic or acute disease; patients taking drugs that could affect the heart; patients with abnormal EF, valvular disease, regional hypokinesia on echocardiography; and individuals involved in competitive sports are excluded.

Operational definition

Obesity was defined as a BMI of >25 kg/m^2^, with clear evidence of excessive subcutaneous adipose tissue on physical examination as per the WHO guidelines for the South Asian population. 

The participants in the study were further classified into three groups using physical examination by the primary investigator who is a trained clinician based on the BMI cut-off points (kg/m^2^): a normal weight (control) group had a BMI of <25 kg/m^2^, the overweight group had a BMI of 23-24.9 kg/m^2^, obesity group had a BMI greater than 25 kg/m^2^ [19].

Echocardiographic assessment

Echocardiography was performed using the iE33, Philips system that was observed to have a sensitivity of the detection of elevated LV filling pressure of 0.81, specificity of 0.86, positive predictive value (PPV) of 0.79, negative predictive value (NPV) of 0.74, and overall accuracy of 0.89, and it was performed using a standardized protocol by a trained sonographer who is blinded to the study [20].

Conventional diastolic function assessment was performed using an apical view and the following parameters were assessed (Tables 1 and 2)

**Table 1:** Parameters to assess diastolic function Diastolic dysfunction was assessed using recent recommendations of the American Society of Echocardiography and the European Association of Cardiovascular Imaging 2016 [20]. LVIDd, left ventricular internal diameter at end diastole; IVSd, diastolic interventricular septal thickness; EDV, ejection diastolic volume; E/A, early diastolic filling velocity/late diastolic filling velocity; é, mitral annular velocity; IVRT, iso-volumetric relaxation time

Serial No	Parameters	Units
1	LVIDd	cm
2	IVSd	cm
3	EDV	ml
4	E/A ratio	-
5	E/é	-
6	IVRT	ms

**Table 2:** Parameters to assess systolic function ESV, ejection systolic volume; IVSs, systolic interventricular septal thickness; EF, ejection fraction; LVIDs, left ventricular internal diameter at end systole

Serial No	Parameters	units
1	ESV	ml
2	IVSs	cm
3	EF	%
4	LVIDs	cm

Statistical analysis

For statistical analysis, SPSS V.22.0 software (SPSS, Chicago, IL, USA) was used. The results for categorical variables were presented as numbers and percentages. Continuous variables were presented as mean and standard deviation (SD). We evaluated the accuracy of our echocardiographic system in detecting LVDD based on its sensitivity, specificity, PPV, and NPV. Pearson’s correlation analysis was used to examine the associations between BMI and echocardiographic parameters of LVDD. To compare echocardiographic parameters among three groups of obese patients, variance analysis using one-way ANOVA was used for continuous variables, and post hoc analysis was carried out using Tukey’s HSD test. A P value of <0.05 was considered to indicate a statistically significant result.

## Results

The measured demographic variables of left ventricular systolic and diastolic function in the case and control groups are displayed in Table 3. The volume indices of systolic and diastolic function (ejection diastolic volume (EDV) and ejection systolic volume (ESV)) and iso-volumetric relaxation time (IVRT) were found to be significantly higher in obese patients while relative thickness of the wall and internal diameter was almost comparable to the control group. In obese individuals, significantly lower values of contractility indices were reported such as ejection percentage, early diastolic filling velocity and late diastolic filling velocity (E/A) ratio, and mitral annular velocity as compared to the control group.

**Table 3:** Shows measured echocardiography indices for study groups A P value of <0.05 is considered as significant. BMI, body mass index; ESV, ejection systolic volume; IVSs, systolic interventricular septal thickness; EF, ejection fraction; LVIDd, left ventricular internal diameter at end diastole, LVIDs, left ventricular internal diameter at end systole; IVSd, diastolic interventricular septal thickness; EDV, end diastolic volume; E/A, early diastolic filling velocity/late diastolic filling velocity; é, mitral annular velocity; IVRT, iso-volumetric relaxation time; SD, standard deviation

Variables	Group A Obesity (n=40) Mean±SD	Group A1 Overweight (n=35) Mean±SD	Group B Control (n=75) Mean±SD	P Value
Age (Years)	42.45±14.15	40.55±13.65	43.30±10.70	0.714
Gender	Male/Female	Male/Female	Male 39 (52%)/Female 36 (48%)	
BMI (kg/m^2^)	28.08±4.15	25.25±4.40	22.32±5.62	0.01*
ESV (ml)	44.58±3.96	42.88±5.75	41.58±6.50	0.01*
IVSs (cm)	1.25±0.02	1.15±0.35	1.10±0.35	1.00
EF%	56.86±5.19	60.81±6.40	63.81±7.30	0.01*
LVIDs (cm)	3.02±0.48	3.20±0.86	3.31±0.94	0.06
LVIDd (cm)	4.62±0.75	4.65±1.05	4.69±0.96	0.351
IVSd (cm)	0.94±0.02	0.95±0.27	0.96±0.27	0.714
EDV (ml)	107.44±14.77	104.20±11.20	98.22±12.50	0.002*
E/A Ratio	0.81±0.20	0.98±0.15	1.24±0.25	0.001*
E/é	6.80±1.17	7.30±2.12	8.36±1.54	0.001*
IVRT (ms)	109.84±13.91	101.79±12.03	92.79±11.30	0.001*

Post hoc analysis among the three groups found no significant difference among echocardiographic parameters of the obesity and overweight groups (P>0.05) (Table 3).

Correlation of BMI and echocardiography parameters

**Figure 1:**
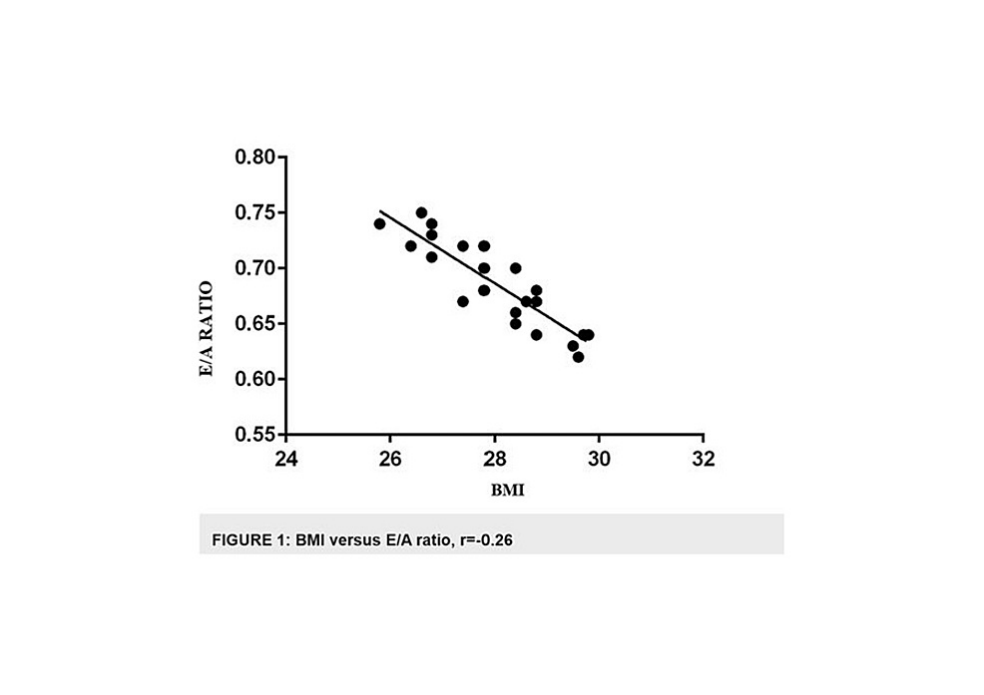
BMI versus E/A ratio

A Pearson bivariate correlation test was used to assess the echocardiographic parameters and BMI. The mitral inflow E/A ratio was shown to have a negative correlation with BMI (r=0.26, P=0.0166), but no other metrics were found to be significantly correlated. Left atrial (LA) diameter showed a moderate relationship in both systole and diastole (r=0.48, P=0.0001; r=0.35, P=0.0005) (Table 4, Figure 1)

**Table 4:** Shows the correlation of BMI with the echocardiography parameters BMI, body mass index; ESV, ejection systolic volume; EF, ejection fraction; LVIDs, left ventricular internal diameter at end systole; LVIDd, left ventricular internal diameter at end diastole; IVSd, diastolic interventricular septal thickness; EDV, end diastolic volume; E/A, early diastolic filling velocity/late diastolic filling velocity; é, mitral annular velocity; IVRT, iso-volumetric relaxation time

Echo Variables	BMI Coefficient	P Value
ESV (ml)	0.26543	0.106
IVSs (cm)	0.34556	0.252
EF%	0.32145	0.74
LVIDs (cm)	0.77834	<0.001
LVIDd (cm)	0.75631	<0.001
IVSd (cm)	0.34456	0.487
EDV (ml)	0.03456	0.561
E/A ratio	0.45456	<0.002
E/é	0.187654	0.54
IVRT (ms)	0.234453	0.36

## Discussion

Based on mounting evidence of a significant prevalence of cardiovascular disease in Asian countries, the WHO expert group evaluated the BMI cut-off figure of BMI greater than 25 kg/m^2^ for Asian Indians [18]. The results of our investigation demonstrated subclinical alterations in LV function in the obese group (Group A) without overt comorbidities.

Although the obese group had lower levels of the LV morphological indicators LVID and IVSD, the difference was not statistically significant.

Correlational estimations revealed a moderate connection between LA diameter in systole and diastole and BMI (r=0.48, P=0.0001; r=0.35, P=0.0005). Our results are comparable to Wong CY et al. and Kossaify A et al. who showed an alteration in LV morphology [21,22]. However, the results of our study are contradictory to earlier findings by Pascual M et al., Chakko S, and Iacobellis G et al. indicating LV systolic function is intact in people categorized into obesity and overweight [23-25].

The E/A ratio was much lower in obese participants due to an increased late diastolic filling and a mostly stable early diastolic filling velocity. This might be because obese people's LVs tend to relax unusually and depend more heavily on left atrial contraction for appropriate filling [10,22].

In their investigation, Garg et al. discovered that 35% of obese participants and 53% of overweight subjects both had diastolic dysfunction with normal EF [26]. While the results for active mitral filling (A) were not significantly impacted, Rozenbaum Z et al. detected a significant decline in the maximal velocity of the passive mitral filling (E) in obese patients, leading to a fall in the E/A ratio [27].

Contrarily, Chakko et al. did not discover any significant variations in the values of E, but they did discover an increase in the values of A, which led to a decrease in the E/A ratio [24]. Hassan BA et al. discovered a significant rise in both E and A levels, which were unaffected by the E/A ratio since they were positively connected with the proportion of body weight that was above the optimum [28]. According to Turkbey EB et al. and numerous other investigations, the majority of obese persons' LV EF was normal to rise, which illustrates the sensitivity of transmitral flow indices to loading conditions as well as the impact of LV hypertrophy [11].

The methods utilized to evaluate the LV systolic function may be the cause of this disparity. Newer echocardiographic techniques can detect subclinical LV functional alterations in a more sensitive way than older echocardiographic techniques, which include measurements like EF, fractional shortening, and circumferential fiber shortening [29,30].

Reduced mitral annular velocity (Sm), as it was shown in the obese group in our study, is suggestive of early LV systolic dysfunction. It is interesting to note that this discovery has only recently been made in Asian Indians after being previously noted in Caucasians [31,32].

Increased intravascular volume, activation of the sympathetic and renin-angiotensin-aldosterone systems, abnormal production of myocardial growth factors from abdominal and cardiac adipose tissue, and metabolic changes that increase arterial stiffness and peripheral vascular resistances all play a role in the pathophysiological mechanisms proposed to explain the relationship between obesity and LVH [30,25]. The negative effects of obesity on heart structure and function may also be aggravated by chronic pressure overload and elevated central pressure [24].

Early post-mortem investigations in morbidly obese patients revealed that an increase in epicardial fat and LVH together caused excess heart weight [31]. According to later morphological findings, in nearly one-fifth of instances, increasing left ventricular wall thickness was accompanied by thickening of the right ventricular wall [32].

Our study's limitations include not taking into account how lipid-lowering medications can affect left ventricular diastolic function. Additionally, we have not considered the impact of the persistence of obesity and the comorbidities that are linked to it.

## Conclusions

Volumetric alterations and ejection percent varied considerably among patients with higher BMI compared to control participants. The level of obesity will determine how strongly the other echocardiographic indices were affected. We can therefore draw the conclusion that obesity should be taken into account as a risk factor for upcoming cardiovascular events. Our study has limitations as the length of obesity and left ventricular dysfunction did not show the association. To examine cardiovascular morbidities such as heart failure and overall mortality in healthy obese people with changes in cardiac morphology and function, longitudinal investigations are necessary. It is important to determine how weight loss affects cardiac morphology and function.
